# A Pilot Study of Clinicians' Perceptions of Feasibility, Client-Centeredness, and Usability of the Systematic Tailored Assessment for Responding to Suicidality Protocol

**DOI:** 10.1027/0227-5910/a000796

**Published:** 2021-06-30

**Authors:** Jacinta Hawgood, Tamara Ownsworth, Helen Mason, Susan H. Spence, Ella Arensman, Diego De Leo

**Affiliations:** ^1^ Australian Institute for Suicide Research and Prevention, World Health Organization Collaborating Centre for Research and Training in Suicide Prevention, School of Applied Psychology, Griffith University, Brisbane, QLD, Australia; ^2^ School of Applied Psychology and Menzies Health Institute Queensland; Griffith University, Brisbane, QLD, Australia; ^3^ School of Public Health, College of Medicine and Health, University College Cork, Ireland; ^4^ National Suicide Research Foundation, Cork, Ireland

**Keywords:** suicide risk assessment, psychosocial assessment, client-centered, screening

## Abstract

**Abstract.** *Background:* The Systematic Tailored Assessment for Responding to Suicidality (STARS) is a client-centered, psychosocial needs-based assessment protocol. This semistructured interview obtains client prioritized indicators that contribute to suicidality and informs commensurate care responses for preventing suicide. *Aim:* To pilot the feasibility, client-centeredness, and usability of the STARS protocol, including clinicians' perceptions of ease of use; content validity; and administration within the community setting. *Method:* A convenience sample of clinicians who undertook assessment and/or intervention with suicidal persons and had used STARS between mid-2016 and early 2017 completed an online survey assessing feasibility, client-centeredness, and usability of STARS. *Results:* Of the 51 clinicians who entered the survey, 42 (82.3%; aged 25–74; 69% female) completed it. Overall, perceptions of feasibility and usability of STARS were positive, particularly regarding client-centeredness of the protocol and confidence in information obtained for screening suicidality and informing needs-based priority responses. *Limitations:* The pilot findings are limited by the use of a small convenience sample and the low completion rate of clinicians with STARS training. *Conclusion:* STARS was perceived as a feasible and useful psychosocial needs-based assessment protocol. Suggestions for improving STARS, training requirements, and application to diverse populations are outlined.

Suicide is a multifaceted phenomenon, with multiple contributing factors (World Health Organisation [WHO], 2014). Clinicians working in suicide prevention are faced with the challenge of gauging individuals' suicidality and ensuring their safety. Many suicide risk assessment tools are available; however, there is little evidence to support their validity in terms of predicting suicide (Large et al., 2016; Runeson et al., 2017; Steeg et al., 2018). Consequently, there remains an ongoing struggle to establish an optimal approach for identifying persons likely to suicide and provide commensurate suicide prevention management.

Traditionally, suicide risk was perceived as a “categorical,” stratified outcome of an assessment process reflecting a person's suicidal state (Hawgood & De Leo, 2016). Categories of risk severity are derived from self-report scales comprising well-established population-based risk factors (Carter et al., 2017; Franklin et al., 2017). However, since Pokorny's (1983) landmark study, which demonstrated the fallibility of these scales to reliably predict suicide, and subsequent consistent findings (Carter et al., 2017; Quinlivan et al., 2017; Runeson et al., 2017), there has been a move from “risk prediction” toward psychosocial needs-based assessment (Carter et al., 2016) using client-centered approaches that seek self-narratives of the suicidal state to inform management responses (Michel & Jobes, 2011). Traditional scales typically do not measure psychosocial risk and protective factors. Clinical practice guidelines in the United Kingdom and Australia now recommend psychosocial needs-based assessment approaches as the gold standard of care (National Collaborating Centre for Mental Health, 2004; Carter et al., 2016). Thus, holistic needs-based assessment of the person's life circumstances, formulation, and safety planning are integral to informing management plans.

Evidence for reductions in suicidality has been found for person-centered approaches: for example, Collaborative Assessment and Management of Suicidality (Jobes, 2006) and Attempted Suicide Short Intervention Program (Michel & Gysin-Maillart, 2015). These approaches emphasize the therapeutic alliance, engaging clients in telling their story around suicidal experiences, and are inclusive of suicide-specific treatment. However, administration of such treatment is not always feasible nor within the remit of many community-based mental health organizations who primarily assess and refer to specialist services. Furthermore, psychosocial assessment should systematically assess both risk *and* protective factors, which are critical for engaging in safety planning and reducing the potential for suicide (Stanley & Brown, 2012). To meet the needs of Australian community-based organizations, the Systematic Tailored Assessment for Responding to Suicidality (STARS; Hawgood & De Leo, 2016) assesses multiple psychosocial risk *and* protective factors from the client's perspective to inform safety planning and commensurate actions.

The STARS protocol does not attempt to predict suicide; rather, it employs a structured professional judgment approach (Cramer & Kapusta, 2017) with empirically informed questions regarding suicidal state (Part A), risk (Part B), and protective factors (Part C) to inform care responses (see Figure S1 in ESM 1). Instead of stratified risk levels, STARS includes client-rated “levels of concern” associated with Parts A and B, and client's narrative and therapist judgment are integrated to guide priority areas for safety planning and management responses.

This pilot study examines the feasibility, client-centeredness, and usability of STARS (2015 Edition), for which training on use of the protocol was available, but not required for its use. It also examines the impact of STARS training upon clinicians' perceptions of utility given that suicide prevention training has been found to impact positively on clinicians' knowledge, skills, and attitudes toward suicide prevention (Pisani et al., 2011; Yonemoto et al., 2019).

### Study Aims

For suicide assessment protocols to have utility in clinical practice, these need to be viewed by clinicians as feasible for administration and useful in terms of information gained regarding clients' suicidality and management responses. The primary aim was to pilot the feasibility, client-centeredness, and usability of STARS (Hawgood & De Leo, 2015 Edition) with respect to clinicians' real-world administration. A secondary aim was to examine associations between clinicians' demographic and work-related characteristics, including STARS training, and their perceptions of the protocol. It was hypothesized that clinicians who had undertaken STARS training would have more favorable perceptions of STARS. A final aim was to elicit clinicians' feedback on contextual issues and how the protocol could be refined to improve its utility.

## Method

### Participants and Design

The sample included Australian clinicians working with suicidal persons who had undertaken some form of suicide prevention training, had utilized STARS (2015 Edition) between June 2016 and February 2017, and volunteered to participate. Of 51 participants who commenced the online survey, 42 (82.3%) completed sufficient sections for the analysis (i.e., at least 90%), whereas nine participants only completed demographic questions. Chi-square tests indicated no significant differences in demographic or work characteristics between those who did (*n* = 42) and did not complete the survey (*n* = 9).

Of the 42 participants, 69% were female. Participants were aged 25–79 years with most aged 30–49 years (73.4%). Health professionals comprised 85.7% of the sample; most had a master's degree (57.14%); over one-third had worked with suicidal persons for >10 years (38.1%) or for 4–7 years (38.1%). See Table S1 in ESM 2 for further details.

### Measures

The survey included sections on sociodemographics (12 questions) and STARS administration and utilization (16 questions plus an item on future development of STARS). Rating scales with 7- or 5-point Likert scales and open-ended questions were included. For example, items assessed ease of administration (1 = *extremely difficult*, 7 = *extremely easy*), confidence in information from STARS (1 = *not at all confident*, 7 = *extremely confident*), perceptions of STARS as a client-centered tool (1 = not at all client-centered, 5 = *very client-centered*), and effectiveness of STARS (1 = *not effective*, 5 = *extremely effective*). Perceptions regarding contextual elements of STARS administration were measured by a “yes/no” format with optional open-ended comments (see overview in ESM 3).

### Procedure

The study was approved by the Griffith University Human Research Ethics Committee (2016/944/HREC), and participants indicated consent by proceeding with the survey. No incentives or reimbursements were offered. Study advertisements targeted clinicians working in suicide prevention using the same multimedia platforms (e.g., Facebook and Twitter) used for dissemination of the STARS protocol. Only those who had used STARS were eligible to participate (STARS trained or not). The survey was completed anonymously on-line (20–25 min).

Recruitment of approximately 30–50 professionals was planned as appropriate to the exploratory nature of the study (Daniel, 2012).

### Data Analysis

Data were screened for accuracy of entry and missing data. Little's Missing Completely at Random test indicated that data were missing at random (χ^2^_(78)_ = 92.10, *p* = .131). Listwise, deletion was used for analyses; hence, the sample size varied between *n* = 37 and *n* = 42 (Table 1). Thirty-three participants (79%) completed one or more open-end questions. Initial analyses involved review of descriptive data and testing for assumptions. Nonparametric statistical tests (χ^2^; Fisher's exact test for <5 cell size) were used due to the small sample size and nominal data. For ease of analysis and due to the modest sample size, age and years of experience were dichotomized using a median split (i.e., age <45 vs. ≥45 years; years of experience in working with suicidal persons <7 vs. ≥7 years). Responses to open-ended questions were coded using a content analysis with similar responses clustered to depict the frequency of responses. Two authors (J.H./M.H.) were involved in (1) reviewing all free-text responses; (2) categorizing responses at a broad level into positive, neutral and negative; and (3) clustering responses with similar meaning (e.g., *positive clinician and client administration experience*). Any differences were discussed and finalized with mutual agreement.

**Table 1:** Distribution of participants' responses regarding the STARS Protocol

Items	Clinician perceptions of STARS protocol							
	Not easy to administer					Easy to administer		
	1	2	3	4	5	6	7	*n*
Ease of administration	Extremely difficult	Moderately difficult	Slightly difficult	Neither easy nor difficult	Slightly easy	Moderately easy	Extremely easy	
*n* (%)	0 (0%)	2 (4.88%)	14 (34.15%)	4 (9.76%)	4 (9.76%)	16 (39.02%)	1 (2.44%)	41
	Invalidated and not understood					Validated and understood		
	1	2	3	4	5	6	7	
Perceptions of client feeling validated	Extremely invalidated and misunderstood	Moderately invalidated and misunderstood	Slightly invalidated and misunderstood	Neither validated/understood nor invalidated/ misunderstood	Slightly validated and understood	Moderately validated and understood	Extremely validated and understood	
*n* (%)	0 (0%)	0 (0%)	3 (8.11%)	8 (21.62%)	5 (13.51%)	8 (21.62%)	13 (35.14%)	37
	No confidence/lacking confidence					Confident		
	1	2	3	4	5	6	7	
	Not at all confident	Moderately lacking confidence	Slightly lacking confidence	Neither confident nor lacking confidence	Slightly confident	Moderately confident	Extremely confident	

Confidence in data for screening of suicidality, *n* (%)	1 (2.50%)	0 (0%)	0 (0%)	4 (10.00%)	2 (5.00%)	25 (62.50%)	8 (20.00%)	40
Confidence in data for informing needs-based priority areas, *n* (%)	0 (0.00%)	0 (0%)	0 (0%)	4 (10.00%)	7 (17.50%)	20 (50.00%)	9 (22.50%)	40
	Not client-centered			Client-centered				
	1	2	3	4	5			
Perceptions of effectiveness of STARS as a client-centered tool	Not at all client-centered	Not very client-centered	Undecided on client-centeredness	Moderately client-centered	Very client-centered			
*n* (%)	0 (0.00%)	1 (2.38%)	3 (7.14%)	9 (21.43%)	27 (64.29%)			40
	Not effective			Effective				
	1	2	3	4	5			
	Not effective at all	Slightly effective	Moderately effective	Very effective	Extremely effective			
Perceptions of part A, *n* (%)	0 (0.00%)	1 (2.63%)	5 (13.16%)	22 (57.90%)	10 (26.32%)			38
Perceptions of part B, *n* (%)	0 (0.00%)	1 (2.63%)	5 (13.16%)	22 (57.90%)	10 (26.32%)			38
Perceptions of part C, *n* (%)	0 (0.00%)	2 (5.26%)	9 (23.70%)	21 (55.26%)	5 (13.16%)			37

## Results

Overall, the responses tended to be negatively skewed, reflecting a higher proportion of responses endorsing positive opinions (Table 1). Consequently, the data were recoded into dichotomous outcomes by merging response categories reflecting negative or neutral opinions and those reflecting more positive opinions (Table 1). For example, for ease of administration (7-point Likert scale), responses for 1 = *extremely difficult* to 5 = *slightly easy* were merged into “not easy to administer,” whereas the responses for 6 = *moderately easy* and 7 = *extremely easy* were merged into “easy to administer.” Similarly, items with 5-point Likert scales were recorded by merging negative or neutral response categories (1 = *not effective at all* to 3 = *moderately effective*) and positive response categories (4 = *very effective* and 5 = *extremely effective*).

Based on the recoded data, just under half (41.5%) of clinicians perceived STARS to be easy to administer, whereas over half (56.8%) perceived that clients felt validated and understood. Most clinicians were confident in data from STARS for screening of suicidality (82.5%) and for informing needs-based priority areas (72.5%). The majority also perceived that STARS was client-centered (85.7%) and that the key sections were effective for systematically exploring the suicidal state (Part A; 84.2%), psychosocial risk factors (Part B; 84.2%), and protective factors (Part C; 68.4%).

Chi-square tests were conducted to identify demographic and work characteristics associated with clinicians' perceptions of the feasibility, client-centeredness, and usability of STARS (see Table S2 in ESM 4). Clinicians' perceptions of STARS did not significantly differ by the participation in STARS training (*p* > .05). There were no significant associations between demographic and work characteristics and clinicians' perceptions of STARS (*p* > .05).

## Contextual Issues and Clinician Feedback Regarding STARS Administration

Contextual issues regarding STARS administration refer to the “hands-on” application or how clinicians used the protocol in practice. Regarding order of administration, 42% indicated they typically followed the standard sequence (i.e., A, B, and C), whereas a higher proportion (57.9%) indicated they varied the order or switched flexibly between sections. For the clinical notes section, approximately two-thirds of participants (66.7%) reported that this section assisted in identifying clients' psychosocial needs, and guided documentation of data from prior sections of the protocol. Furthermore, over 70% reported that they used the clinical notes section for documenting collaboration with other professionals (72.5%), and this section encouraged them to document that they had obtained supervision/peer mentoring for the protocol administration (71.8%).

Analysis of responses (*n* = 33) to the open-ended questions indicated that most clinicians (80.6%) had positive experiences of administering STARS. These included comments relating to ease of administration (*n* = 2; e.g., “Implementation was straightforward and easy to administer within a session”), usefulness of STARS for eliciting information (*n* = 7; e.g., “It provided structure and a guiding tool for seeking client answers to difficult questions”), the structured nature of the interview (*n* = 8; e.g., “Great way to structure important areas of questions, while asking and reporting on the client's story/feedback/answers”), and provision of a comprehensive framework for exploring suicidality and eliciting the client's narrative (n = 8; e.g., “Perfect tool for guiding questions that are critical for uncovering person's real pain. Great to hear a story from client which can be used – documenting that story as evidence of the pain and needs to be met.”).

However, a small proportion of participants (19.4%) reported neutral (*n* = 3) or negative experiences (*n* = 3). Such comments typically related to the length of the protocol: “It is a long document if I'm only exposed to a short amount of time per client. On the flip side, I can extract a lot more information about the person and their psycho-social stress/situation at the same time as doing a risk assessment.”

Over half of participants (58%) emphasized the collaborative and client-centered approach of STARS. For example, “STARS allows the client to tell a story in response to each item and then they watch as you write it down … it seems to make them feel that you truly see them as important.” Two reported that while STARS was initially difficult to administer, training and experience with the protocol had improved their capacity to remain client-centered throughout the assessment.

Of the total sample, 55% (*n* = 23) participants provided open-ended feedback on Part A. Several noted that questions were effective (*n* = 5) and comprehensive (*n* = 4), encouraged clients to disclose their experiences (*n* = 3), provided a good/detailed overview of the client's current suicidality (*n* = 4), and yielded better information than other tools they had used (*n* = 2). However, three commented that questions were omitted about medication use and self-criticism, and guidance on how to ask about intent. One participant suggested simplifying the format, and another recommended providing key questions to explore agitation, numbness, and dissociation.

For Part B, 23% (*n* = 10) provided open-ended feedback. Some participants provided more than one comment including that the section contained appropriate content (*n* = 4), that items were framed within the client-centered approach (*n* = 2), and that the information gained was better than that of other tools (*n* = 1). However, some participants indicated that Part B was missing important risk factors, including gender-related issues (*n* = 2), LGBTIQ+ (*n* = 2), bullying (*n* = 1), interpersonal conflict (*n* = 1), postnatal depression (*n* = 1), and sexual abuse (*n* = 4).

For Part C, 36% (*n* = 15) provided open-ended comments. Positive feedback included that such information was often missed in risk assessment (*n* = 6) and how protective factors are important for safety planning and management (*n* = 4). Nevertheless, others noted difficulty in obtaining this information (*n* = 4), which was attributed to their clients' struggle to recognize these as follows: “It's hard to get positive comments from people who are in a really low point especially if they also have severe depression and PD.” One participant indicated that the relevance of Part C items varied according clients' access to and connection to their community.

Of the total sample, 62% (n = 26) provided open-ended comments on the clinical notes section. Most participants (*n* = 16) made comments indicating that this section was straightforward and provided a good summary of key information (e.g., “It made me take all the client answers into a summarised picture”). However, some commented that the section was lengthy (*n* = 2), involved a lot of writing (*n* = 3), and more space was needed for writing (*n* = 3) and integrating information from other safety planning tools (*n* = 1). One participant reported that this section would be easier to complete if they were more familiar with STARS.

Of the total sample, 57.1% (*n* = 24) provided suggestions on the future development of STARS (some more than one comment). Comments included desire for a “short-form” (*n* = 5) or electronic version or interactive PDF (*n* = 2) of the protocol. Others suggested providing more space to record client responses verbatim (*n* = 4), having a Mental State Examination section at the beginning of the protocol (*n* = 1), including physical health or illness in Part B (*n* = 1) and developing a client follow-up section (*n* = 1). Recommended adaptations of STARS for specific populations and settings included prison (*n* = 2), youth (*n* = 7), Indigenous Australians (*n* = 3), LGBTIQ+ (*n* = 2), and veterans and/or post-traumatic stress disorder (*n* = 3). Translation of STARS to other languages (*n* = 2) and greater access to STARS specific training (*n* = 2) were also suggested.

## Discussion

This study sought clinicians' perceptions of the STARS protocol with the primary aim of piloting its feasibility, client-centeredness, and usability in practice. Undertaking feasibility testing of this type is essential to inform ongoing refinement of the protocol. Overall, perceptions were largely favorable, with most participants considering STARS to be effective as a client-centered tool and having confidence in the information gained to screen suicidality and inform needs-based priority responses. The majority of clinicians also provided positive feedback on the effectiveness of key sections for systematically exploring suicidal state, psychosocial risk, and protective factors.

Nonetheless, less than half of the participants perceived STARS to be easy to administer. The feedback indicated that this was partly due to the lengthy process of administration (duration 1–1.5 h). Although responses were not significantly related to training status, given that less than one-third of the sample had undertaken STARS training, it is likely that most clinicians received little guidance on processes and skills needed to enhance ease of administration. Several participants recommended that training specifically on STARS be more available or requested greater guidance for sections of the protocol (e.g., Part A). Importantly, most participants had high confidence in the data obtained from all sections of STARS, supporting that these meet the purpose for which they were designed. These preliminary findings provide valuable information concerning the real-world application and suggested enhancements of STARS.

The second aim was to identify sociodemographic and work-related characteristics related to perceptions of feasibility, client-centeredness, and usability of STARS. Given that STARS training was not mandatory for use of the protocol at the time of the survey, we expected that those who had undertaken STARS training would have more positive perceptions of the protocol. However, contrary to our expectations, there were no differences in perceptions according to their STARS training status. A possible reason for this finding is that STARS training originally focused broadly on education regarding suicide prevention/management, rather than STARS administration specifically. Although the protocol included guidelines for administration, it was accessible to all users, regardless of them having received STARS training. Furthermore, years of experience and recency of training were not significantly associated with clinicians' perceptions. Caution is warranted in interpreting these findings due to the small sample size, which may have affected statistical power.

## Limitations

In addition to small sample size, a key limitation relates to the use of volunteers from limited recruitment sources and self-selection of the sample. Use of a convenience sample combined with small sample size may affect the representativeness of the sample and bias the findings in terms of gaining only the perceptions of clinicians who were motivated to complete the survey. Recruitment of a volunteer sample via multimedia platforms may have limited access to the survey for clinicians using STARS who were not connected to these platforms. A further limitation relates to potential memory bias due to the retrospective recall involved in answering questions about STARS administration, which may have affected the accuracy of responses. Furthermore, minimal information was sought regarding participants' work characteristics and experience with using STARS. Information regarding the frequency of use and assessments conducted with STARS, factors influencing the use of the protocol (e.g., nature of client presentation), and contexts for use (e.g., acute psychiatric vs. community setting) may have been informative for understanding clinicians' perceptions.

Future research should employ a more representative sampling approach and a larger sample and focus specifically upon clinicians who have received STARS training. Where probability sampling is not feasible nor suited to the research objective, the use of “homogenous convenience sampling” has been recommended (Jager et al., 2017). Findings derived from this sampling approach have arguably clearer generalizability than heterogenous convenience sampling as employed in the current study (Bornstein et al., 2013). Future studies might also compare clinicians' perceptions of STARS with their views on other person-centered protocols. It is also recommended that independent evaluation of STARS occurs by researchers not involved in developing the protocol.

Since receiving feedback from the current study, a 2-day training program has been introduced (Hawgood & De Leo, 2018) for STARS administration with a skills and techniques focus. Originally, STARS training was 1-day noncompulsory (from 2015 to 2017). Training is now mandated for using STARS in practice. Furthermore, clients' perspectives on their experience of STARS administration would be valuable.

## Conclusion

Overall, perceptions of STARS as a client-centered, psychosocial needs protocol were largely positive regarding confidence in data obtained, and perceptions of key sections as effective. The need for training on administration was highlighted, and adaptations for specific populations were suggested. Accordingly, a mandated 2-day training to support administration now exists. Feedback from this study will be incorporated into future editions of STARS; some aspects are evident in the interim 2018 Edition. Further research is needed to evaluate psychometric properties of STARS, client experiences, and the impact of training on clinicians' competency.

## Electronic Supplementary Material

The electronic supplementary material is available with the online version of the article at https://doi.org/10.1027/0227-5910/a000796

**ESM 1.** Figure S1 with STARS protocol approach
**ESM 2.** Table S1 with demographic characteristics of clinicians who commenced the survey
**ESM 3.** Overview of the clinician survey
**ESM 4.** Table S2 with distribution of clinician responses according to demographic and work characteristics
