# Supplementary figures and images for: A Pilot Study of Clinicians' Perceptions of Feasibility, Client-Centeredness, and Usability of the Systematic Tailored Assessment for Responding to Suicidality Protocol
Source: Crisis. 2021 Jun 30;43(6):523–30. doi: 10.1027/0227-5910/a000796 (PMC9716345; doi:10.1027/0227-5910/a000796)

## Electronic Supplementary Material 1

**Figure S1.** STARS Protocol Approach: Sections and Items

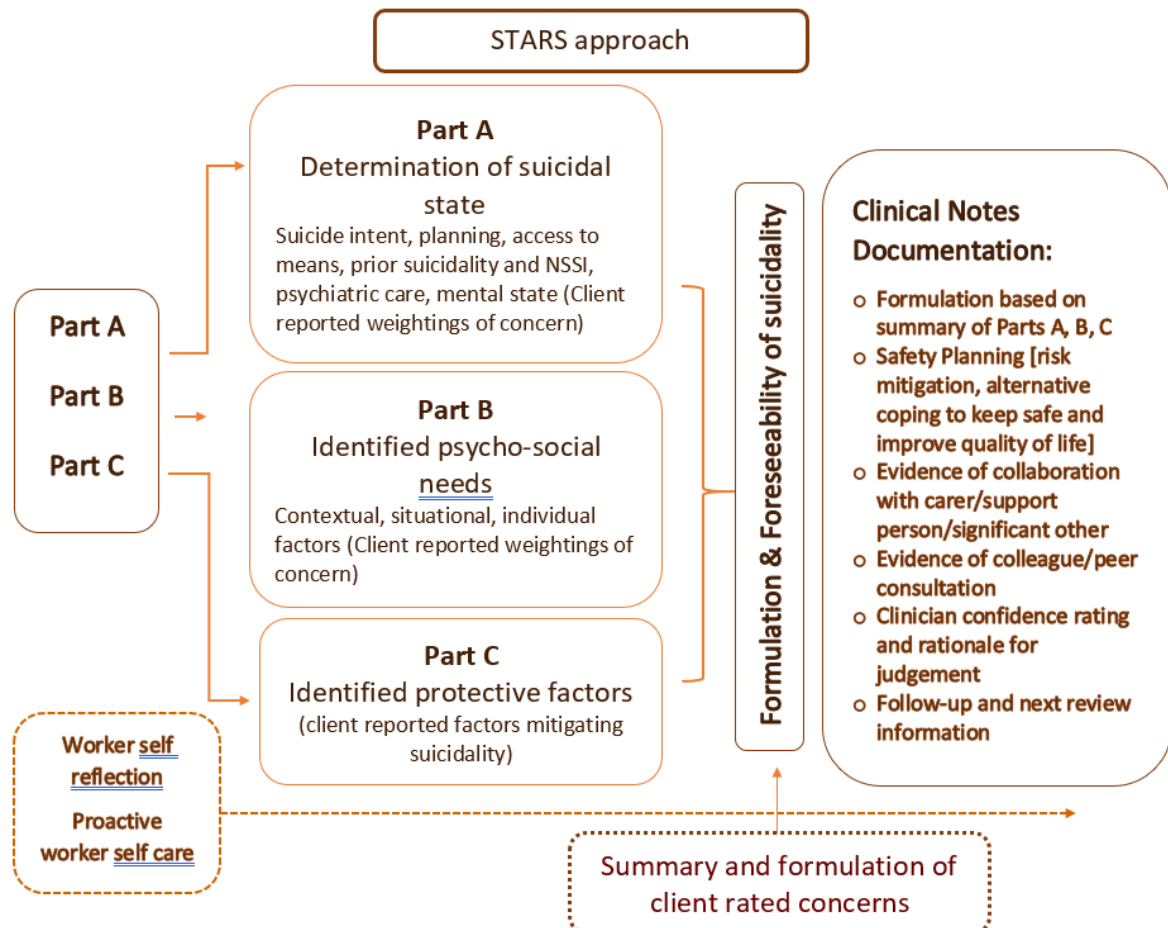

Supplement: Supplementary file 1 [file cri_43_6_523_esm1.pdf]
