# Supplementary material for: A Pilot Study of Clinicians' Perceptions of Feasibility, Client-Centeredness, and Usability of the Systematic Tailored Assessment for Responding to Suicidality Protocol
Source: Crisis. 2021 Jun 30;43(6):523–30. doi: 10.1027/0227-5910/a000796 (PMC9716345; doi:10.1027/0227-5910/a000796)
Supplement: Supplementary file 2 [file cri_43_6_523_esm2.pdf]

https://doi.org/10.1027/0227-5910/a000796

Electronic Supplementary Material 2

Table S1. *Demographic Characteristics of Clinicians who Commenced the Survey*

		Completed	Did not Complete	*n*
Gender				
	Male	13	1	14
	Female	29	8	37
		42	9	
Age				
	<45years	23	5	28
	≥45years	19	4	23
Work Status				
	Full time	23	5	28
	Part time	12	4	16
	Casual	7		7
Worker Role[a]				
	Health Professional	36	6	42
	Non-Health Professional	6	3	9
Level of Education				
	Undergraduate degree	5	2	7
	4th Year degree	9	1	10
	Masters degree	24	6	30
	PhD	4		4
Years working with suicidal persons				
	<7 years	19	4	23
	≥7years	23	5	28
Completed STARS Training				
	Yes	12	1	13
	No	30	8	38
Recency of Training				
	<12months	26	3	29
	≥12months	16	6	22
Recency of Supervision				
	<12months	33	5	38
	≥12months	9	4	13

[a] Health professionals had qualifications in an allied health or health occupation (e.g., psychologists, social worker, nurse, doctor, occupational therapist), whereas non-health professionals had qualifications or experiences in community welfare, youth work, volunteer community work, or other human service roles.
