# Supplementary material for: A Pilot Study of Clinicians' Perceptions of Feasibility, Client-Centeredness, and Usability of the Systematic Tailored Assessment for Responding to Suicidality Protocol
Source: Crisis. 2021 Jun 30;43(6):523–30. doi: 10.1027/0227-5910/a000796 (PMC9716345; doi:10.1027/0227-5910/a000796)
Supplement: Supplementary file 3 [file cri_43_6_523_esm3.pdf]

https://doi.org/10.1027/0227-5910/a000796

Electronic Supplementary Material 3

Overview of the Clinician Survey

The specific breakdown of survey items and their content areas is as follows:

Survey Part A – Participant demographic variables: 12 questions sought information on location of residence, age, gender, position/role and working status, qualifications/education, years of experience working with suicidal persons, and training and supervision experiences in suicide risk assessment. Two of these 12 items including an additional free-text area for any additional comments (e.g. please specify what type of training).

Survey Part B – STARS administration and utilisation: 16 questions sought professionals' perceptions of the feasibility, utilisation, and contextual elements of administration of STARS with clients. All of these items included a free-text area to allow for any additional perceptions or comments (e.g., please comment; why/why not?). Finally, we included an additional stand-alone item, an open-ended question, at the very end of the survey, that sought ideas for future development, design and application of the STARS protocol for different client populations, resulting in a total of 17 items for Part B of the survey.

Part B survey items included both quantitative and qualitative information (free-text items). The quantitative items included a combination of categorical variables (8 items), and continuous variables (8 items) which were 7- and 5-point Likert Scales (4 items and 4 items, respectively). For example, the 7-point Likert scale for ease of administration ranged from 1 (extremely difficult) to 7 (extremely easy); and the scale for client experience of STARS administration ranged from 1 (extremely displeased - totally misunderstood and invalidated) to 7 (extremely pleased – totally understood and validated). Two further questions measured confidence in data from STARS for screening for suicidality and informing needs-based priority areas (1 = not at all confident, 7 = extremely confident). To measure perceptions of

https://doi.org/10.1027/0227-5910/a000796

effectiveness of STARS as a client-centred tool and effectiveness of the STARS protocol including Parts A, B and C, a 5-point Likert Scale was used (1 = not effective to 5 = extremely effective). Perceptions about the contextual elements of administration of STARS were measured by a dichotomous 'yes/no' response format with an option for open-ended comments in free text boxes. For example, 4 items asked respondents about views on the Clinical Notes section and its use for supporting consultation with peers and guiding documentation of summary data. Another 3 items asked about perceptions of the prioritizing of specific enquiry answers pertaining to Parts, A, B and C sections, and a final dichotomous item asked participants whether they administered STARS in the order of interview sections (i.e., consecutively) or by moving between the different sections.
