# Supplementary material for: A Pilot Study of Clinicians' Perceptions of Feasibility, Client-Centeredness, and Usability of the Systematic Tailored Assessment for Responding to Suicidality Protocol
Source: Crisis. 2021 Jun 30;43(6):523–30. doi: 10.1027/0227-5910/a000796 (PMC9716345; doi:10.1027/0227-5910/a000796)
Supplement: Supplementary file 4 [file cri_43_6_523_esm4.pdf]

https://doi.org/10.1027/0227-5910/a000796

Electronic Supplementary Material 4

Table S2. Distribution of Clinician Responses According to Demographic and Work Characteristics[1]

Sample characteristics	Ease of administration *n* (%)		Client felt validated *n* (%)		Confidence for screening of suicidality *n* (%)		Confidence for informing priority areas *n* (%)		Client-centred approach *n* (%)		Effectiveness of STARS Part A		Effectiveness of STARS Part B		Effectiveness of STARS Part C	
	**Yes**	**No**	**Yes**	**No**	**Yes**	**No**	**Yes**	**No**	**Yes**	**No**	**Yes**	**No**	**Yes**	**No**	**Yes**	**No**
**Gender**																
Female	13 (44.8)	16 (55.2)	16 (57.1)	12 (42.9)	24 (85.7)	4 (14.3)	20 (71.4)	8 (28.6)	26 (92.9)	2 (7.1)	21 (80.8)	5 (19.2)	22 (81.5)	5 (18.5)	19 (70.4)	8 (29.6)
Male	4 (33.3)	8 (66.7)	5 (55.6)	4 (44.4)	9 (75)	3 (25)	9 (75)	3 (25)	10 (83.3)	2 (16.7)	11 (91.7)	1 (8.3)	10 (90.9)	1 (9.1)	7 (70)	3 (30)
**Age**																
< 45 years	7 (30.4)	16 (69.6)	11 (50)	11 (50)	17 (77.3)	5 (22.7)	16 (72.7)	6 (27.3)	20 (90.9)	2 (9.1)	18 (85.7)	3 (14.3)	19 (86.4)	3 (13.6)	16 (76.2)	5 (23.8)
≥ 45+ years	10 (55.6)	8 (44.4)	10 (66.7)	5 (33.3)	16 (88.9)	2 (11.1)	13 (72.2)	5 (27.8)	16 (88.9)	2 (11.1)	14 (82.4)	3 (17.6)	13 (81.3)	3 (18.7)	10 (62.5)	6 (37.5)
**Worker Role**																
Health Professional (HP)	13 (37.1)	22 (62.9)	15 (48.4)	16 (51.6)	28 (80)	7 (20)	25 (71.4)	10 (28.6)	31 (88.6)	4 (11.4)	27 (81.8)	7 (21.2)	26 (81.3)	6 (18.7)	22 (68.7)	10 (31.3)
Non-HP	4 (66.7)	2 (33.3)	6 (100)	0 (0)	5 (100)	0 (0)	4 (80)	1 (20)	5 (100)	0 (0)	5 (100)	0 (0)	6 (100)	0 (0)	4 (80)	1 (20)
**Experience Working with suicidal persons**																
< 7 years	7 (36.8)	12 (63.2)	10 (55.6)	8 (44.4)	17 (94.4)	1 (5.6)	14 (77.8)	4 (22.2)	16 (88.9)	2 (11.1)	17 (94.4)	1 (5.6)	18 (94.7)	1 (5.3)	14 (77.8)	4 (22.2)

[1] None of the chi-square analyses examining differences in clinician responses for any of the items were found for any of the demographic or work characteristics.

https://doi.org/10.1027/0227-5910/a000796

≥7+ years	10 (45.5)	12 (54.5)	11 (57.9)	8 (42.1)	16 (72.7)	6 (27.3)	15 (68.2)	7 (31.8)	20 (90.9)	2 (9.1)	15 (75)	5 (25)	14 (73.7)	5 (26.3)	12 (63.2)	7 (36.8)
**Type of Training**																
STARS trained	7 (58.3)	5 (41.7)	7 (58.3)	5 (41.7)	8 (66.7)	4 (33.3)	8 (66.7)	4 (33.2)	9 (75)	3 (25)	10 (90.9)	1 (9.1)	9 (81.8)	2 (18.2)	9 (81.8)	2 (18.2)
Not STARS trained	10 (34.5)	19 (65.5)	14 (56)	11 (44)	25 (89.3)	3 (10.7)	21 (75)	7 (25)	27 (96.4)	1 (3.6)	22 (81.5)	5 (18.5)	23 (85.2)	4 (14.8)	17 (65.4)	9 (34.6)
**Recency of Training**																
<12 months	10 (40)	15 (60)	9 (40.91)	13 (59.09)	18 (72)	7 (28)	(16) 64	(9) 36	21 (84)	4 (16)	19 (79.2)	5 (20.8)	18 (78.3)	5 (21.7)	14 (63.6)	8 (36.4)
≥12 months	7 (43.75)	9 (56.25)	12 (80)	3 (20)	15 (100)	0 (0)	(13) 86.7	(2) 13.3	15 (100)	0 (0)	13 (92.9)	1 (7.1)	14 (93.3)	1 (6.7)	12 (80)	3 (20)
**Recency of Supervision**																
<12 months	13 (40.63)	19 (59.38)	15 (51.7)	14 (48.3)	25 (78.1)	7 (21.9)	(21) 65.6	(11) 34.4	28 (87.5)	4 (12.5)	24 (80)	6 (20)	23 (79.3)	6 (20.7)	22 (75.9)	7 (24.1)
≥12 months	4 (44.44)	5 (55.55)	6 (75)	2 (25)	8 (100)	0 (0)	(8) 100	(0) 0	8 (100)	0 (0)	8 (100)	0 (0)	9 (100)	0 (0)	4 (50)	4 (50)
